# Comparison of the Incidence of Postoperative Acute Kidney Injury Following the Administration of Remimazolam or Sevoflurane in Elderly Patients Undergoing Total Knee Arthroplasty: A Randomized Controlled Trial

**DOI:** 10.3390/jpm13050789

**Published:** 2023-05-01

**Authors:** Sangho Lee, Hee Yong Kang, Ye Na Ahn, Ann Hee You

**Affiliations:** Department of Anesthesiology and Pain Medicine, Kyung Hee University College of Medicine, Kyung Hee University Hospital, Seoul 02447, Republic of Korea; silzzang15@naver.com (S.L.);

**Keywords:** acute kidney injury, remimazolam, sevoflurane, elderly, total knee arthroplasty, postoperative complications

## Abstract

Background: We evaluated the incidence of postoperative acute kidney injury (AKI) and complications when remimazolam (RMMZ) or sevoflurane (SEVO) were used in elderly patients undergoing total knee arthroplasty. Methods: Seventy-eight participants aged ≥65 were randomly allocated to either the RMMZ or SEVO group. The primary outcome was the incidence of AKI on postoperative day (POD) 2. The secondary outcomes included intraoperative heart rate (HR), blood pressure (BP), total drug administered, emergence time, postoperative complications on POD 2, and hospital length of stay (HLOS). Results: The incidence of AKI was comparable between the RMMZ and SEVO groups. The doses of intraoperative remifentanil, vasodilators, and additional sedatives were significantly higher in the RMMZ group than in the SEVO group. Overall intraoperative HR and BP tended to remain higher in the RMMZ group. The emergence time in the operating room was significantly faster in the RMMZ group; however, the time required for an Aldrete score ≥ 9 was comparable between the RMMZ and SEVO groups. Postoperative complications and HLOS were comparable between the RMMZ and SEVO groups. Conclusion: RMMZ may be recommended for patients who are expected to decrease in intraoperative vital signs. However, stable hemodynamics with RMMZ were not sufficient to influence the prevention of AKI.

## 1. Introduction

Various incidence rates of postoperative acute kidney injury (AKI) have been reported depending on the type of surgery and drugs administered [1,2,3]. When AKI occurs, it not only delays recovery and affects postoperative outcomes but also increases mortality [4]. Risk factors for AKI include male sex, old age, chronic renal disease, heart failure, diabetes, intraoperative bleeding, nutritional status, and hypotension [5,6].

Total knee arthroplasty (TKA) is mainly performed in patients who experience pain and deterioration in quality of life because of knee cartilage wear or rheumatoid arthritis. Most patients are elderly, and the number of patients undergoing TKA increases with increasing life expectancy [7]. Lower limb ischemia and reperfusion occur when a tourniquet is used on the thigh during surgery, and hypotension often occurs because of blood redistribution during reperfusion. Given these hemodynamic instabilities and aging-related risk factors, studies on AKI after TKA are actively being conducted [8,9].

Remimazolam (RMMZ) is a relatively recently approved, intravenously infused, very short-acting benzodiazepine [10,11,12]. Similar to midazolam, RMMZ acts on γ-aminobutyric acid A receptors to induce sedation or anesthesia [13]. In addition, its action time is short and predictable because of its rapid onset and offset. Similar to other benzodiazepines, flumazenil can be administered at the end of anesthesia to reverse the sedative action of RMMZ [14], and studies on its hemodynamic stability have been reported [15,16,17,18]. Because of these advantages, the use of RMMZ has increased.

However, studies on the prevention of postoperative AKI using the hemodynamic stability of RMMZ have not yet been conducted. We hypothesized that the use of RMMZ, which can promote hemodynamic stability during TKA, would reduce the incidence of postoperative AKI compared to the administration of sevoflurane (SEVO), which is an inhalation anesthetic agent used for conventional anesthesia.

## 2. Methods and Materials

### 2.1. Ethics and Study Design

This study evaluated the incidence of postoperative AKI after RMMZ or SEVO during TKA in elderly patients in an assessor-blinded parallel-group randomized controlled study. Ethical approval was obtained from the Institutional Review Board (IRB) of Kyung Hee University Hospital (KHUH 2022-08-066) on 16 September 2022. The trial was registered with the Clinical Research Information Service (CRIS) (No. KCT0007782; registration date: 7 October 2022; principal investigator: Ann Hee You) and conducted in compliance with the Declaration of Helsinki. The study protocol is available from CRIS. Written informed consent was obtained from all study participants, and the study report complied with the Consolidated Standards of Reporting Trials checklist.

### 2.2. Participants

Elderly individuals older than 65 yrs who planned to undergo elective TKA were included in the trial. Preoperative hemodialysis due to renal failure, allergy to any study drugs, revision TKA, second operation on the opposite side of the knee in one hospitalization, simultaneous TKA, American Society of Anesthesiologists (ASA) class ≥ IV, and body mass index >35 kg/m^2^ were excluded. Recruitment began in September 2022 and was terminated in January 2023.

### 2.3. Outcomes

The primary outcome was the incidence of AKI on postoperative day (POD) 2. AKI was evaluated using the Kidney Disease: Improving Global Outcomes criteria and was defined as an alteration of serum creatinine (Cr) level ≥ 0.3 mg/dL until POD 2 [19].

Secondary outcomes included intraoperative blood pressure (BP), heart rate (HR), total intraoperative drugs, amount of fluid infused, estimated blood loss (EBL), and emergence time. The time from the cessation of anesthetic drugs until an Aldrete score ≥9 was reached [20], and the hospital length of stay (HLOS), were recorded. Postoperative complications within POD 2 were evaluated, including postoperative nausea and vomiting (PONV), postoperative urinary retention (POUR), postoperative pulmonary complications (PPC), and delirium. POUR was defined as retention urine ≥ 300 mL on bladder scan [21,22]. PPC was defined as respiratory infection, changes in chest radiographic findings, and new-onset oxygen demand [23].

The correlation between liver function test (LFT) and recovery time was evaluated in the RMMZ group as a subgroup analysis.

### 2.4. Blind and Randomization

On the morning of surgery, the participants were randomly allocated to the RMMZ or SEVO groups using sealed opaque envelopes. With random block sizes and a 1:1 allocation, a computer-generated random allocation sequence was produced using Microsoft Excel 2019. Because of the considerable differences in the technique of study drug administration, the anesthesia providers were not blinded to group allocation; however, the patients and outcome assessors were blinded.

### 2.5. Procedures

After entering the operating room (OR), the patients’ baseline HR, BP, and peripheral O_2_ saturation were measured, and palonosetron (0.075 mg intravenous (IV)) and glycopyrrolate (0.005 mg/kg IV) were administered before anesthesia induction.

In the RMMZ group, 1 mg/mL RMMZ (ByfavoTM) was prepared, and 0.1 mg/kg IV was administered for anesthesia induction. An additional 2 mg IV was administered, if necessary. If loss of consciousness was confirmed, a neuromuscular blocker was given at 0.8 mg/kg IV rocuronium. When neuromuscular block was confirmed, 0.5 µg/kg IV bolus of remifentanil was administered, and tracheal intubation was performed. Anesthesia was maintained at 1–2 mg/kg/h IV RMMZ and adjusted according to the target range of the bispectral index (BIS), which was 30–60. In the SEVO group, 1.5 mg/kg IV propofol was administered for anesthesia induction. An additional 20 mg IV was administered, if necessary. 0.8 mg/kg IV rocuronium and 0.5 µg/kg IV remifentanil were administered before tracheal intubation as the same protocol with the RMMZ group. SEVO was adjusted with reference to the BIS level during anesthesia maintenance.

In both groups, 0.03 mg/kg IV midazolam was administered if the BIS level did not reach the target. Intraoperatively, remifentanil was adjusted to the range of 0.05–0.2 µg/kg/min IV and was maintained at the 20% range of the initial HR and BP in both groups. If the HR and BP were unable to be maintained by remifentanil alone, a beta blocker (esmolol, 0.3 mg/kg IV), a vasodilator (nicardipine, 0.3–0.5 mg IV), or a vasoconstrictor (phenylephrine, 1 mcg/kg IV) was administered. The rate of intraoperative fluid administration was set at 3–4 mL/kg/h IV. Remifentanil administration was discontinued at the time of skin suturing. Within POD 2, IV, patient-controlled analgesia based on fentanyl (12.5 µg/kg IV) was administered in both groups. RMMZ and SEVO were discontinued at the time of splint application. Sugammadex was administered at 2 mg/kg IV to reverse neuromuscular blockade; in the case of the RMMZ group, 0.2 mg IV flumazenil was administered as a reversal agent of RMMZ.

### 2.6. Data Collection and Outcome Assessment

The incidence of AKI was assessed until POD 2 on the basis of the medical records.

In the OR, HR and BP were measured at baseline, both before and after endotracheal intubation, at the beginning of incision, 30 min and 1 h after incision, at lower extremity tourniquet deflation, and in the post-anesthetic care unit (PACU) phase. The total intraoperative amounts of RMMZ, remifentanil, fluid, other infused medications, and EBL were recorded. SEVO was recorded as the mean vol%, which was adjusted during surgery. The emergence time from RMMZ or SEVO stop to eye opening, endotracheal tube extubation, exit from the OR, and time to reach Aldrete score ≥ 9 was measured. The incidence of postoperative hospital complications within POD 2 and postoperative HLOS wase recorded by reviewing the medical records.

### 2.7. Sample Size

According to preliminary research conducted at our institution in patients who underwent TKA, the incidence rates of AKI were approximately 11% and 39% in the propofol- and remifentanil-based total intravenous anesthesia (TIVA) and inhalational groups, respectively. The number of participants in each group was calculated to be 35 based on the G-power analysis performed using pilot study data (exact test, proportions: inequality, two independent groups [Fisher’s exact test]; a priori: computed required sample size of two tails, proportion p1 0.11, proportion p2 0.39, α error 0.1, power 0.8, and allocation ratio N2/N1 1). The total number of patients was 78, with 39 patients in each group, considering a 10% dropout rate.

### 2.8. Statistical Analysis

Data were expressed as numbers (%) or medians (interquartile ranges). Fisher’s exact test or the chi-square test was used to analyze categorical variables. Normality of the distribution of continuous variables was evaluated using the Shapiro–Wilk test. Comparisons between continuous variables with a normal distribution were computed using independent variable t-tests, whereas comparisons between non-normal variables were performed using Wilcoxon rank-sum tests. Spearman’s rank correlation analysis was performed to determine the correlation between the two non-normal continuous variables. Statistical analyses were conducted using the commercial statistical software SPSS (version 22.0; SPSS Inc., Chicago, IL, USA), and two-tailed *p*-Values < 0.05 were considered statistically significant.

## 3. Results

### 3.1. Study Population and Baseline Characteristics

**Figure 1:**
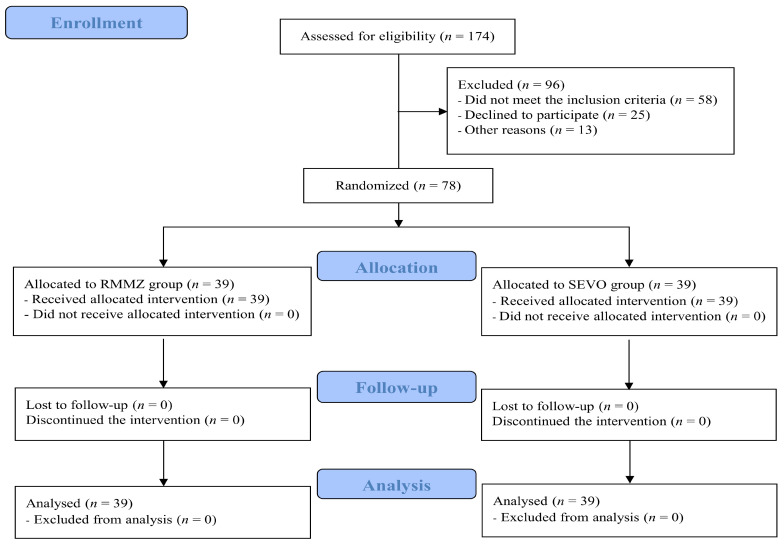
CONSORT diagram of the patient flow chart. RMMZ: remimazolam; SEVO: sevoflurane; CONSORT: Consolidated Standards of Reporting Trials.

A total number of 174 patients were screened; 96 were excluded, and 78 were randomly assigned. Thirty-nine participants were equally allocated to the RMMZ and SEVO groups without any loss to follow-up. Finally, 39 participants from each group were analyzed (Figure 1). Demographic data, medical history, and preoperative laboratory findings were homogeneous between the RMMZ and SEVO groups (Table 1).

### 3.2. Primary Outcome

**Figure 2:**
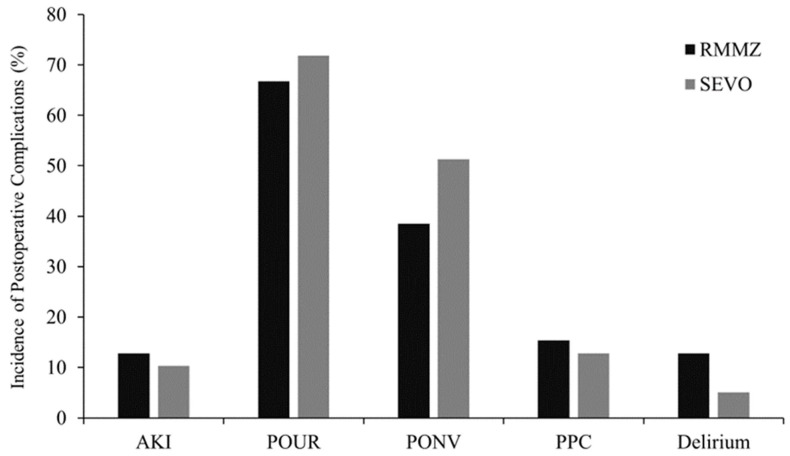
Incidence of postoperative complications. RMMZ: remimazolam; SEVO: sevoflurane; AKI: acute kidney injury; POUR: postoperative urinary retention; PONV: postoperative nausea and vomiting; PPC: postoperative pulmonary complications.

The incidence of AKI was five patients (12.8%, 5/39) in the RMMZ group and four patients (10.3%, 4/39) in the SEVO group on POD 2, with a comparable incidence between the RMMZ and SEVO groups (*p* = 1.000) (Figure 2).

### 3.3. Secondary Outcomes

Anesthesia and surgical times, fluid administration, and EBL were comparable between the RMMZ and SEVO groups. However, the intraoperative remifentanil infusion rate was significantly higher in the RMMZ group (0.11 (0.08–0.14) μg/kg/min IV) than in the SEVO group (0.08 (0.05–0.11) μg/kg/min IV) (*p* = 0.001). The patents received inotropic more frequently in the SEVO group (66.7% (26/39)) than in the RMMZ group (15.4% (6/39)) (*p* < 0.001), and the number of patients receiving nicardipine as vasodilator (84.6% (33/39) vs. 20.5% (8/39), *p* < 0.001) and midazolam as additional sedatives (20.5% (8/39) vs. 0% (0/39), *p* = 0.009) was definitely higher in the RMMZ group than in the SEVO group (Table 2).

**Figure 3:**
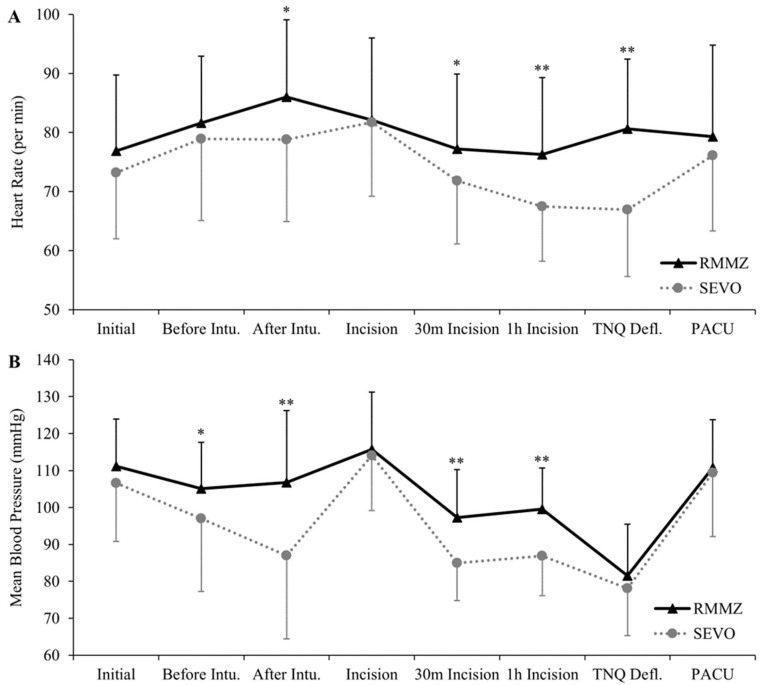
Trends in heart rate and mean blood pressure by different perioperative phages. Each phage was baseline after entering the operating room, before and after endo tracheal intubation, immediate, 30 min and 1 h after skin incision, lower extremity tourniquet deflation at the end of surgery, and after entering PACU. (**A**) Heart rate. (**B**) Mean blood pressure. Data are presented as means ± standard deviations. Significant differences between RMMZ and SEVO group at each point are denoted as * *p* < 0.05 or ** *p* < 0.01. RMMZ: remimazolam; SEVO: sevoflurane; Intu: intubation; TNQ Defl: tourniquet deflation; PACU: post-anesthetic care unit.

Overall, intraoperative HR and BP tended to remain higher in the RMMZ group than in the SEVO group. In particular, HR was significantly higher after tracheal intubation (86.0 ± 13.1/min vs. 78.8 ± 13.9/min, *p* = 0.021), 30 min (77.2 ± 12.7/min vs. 71.8 ± 10.7/min, *p* = 0.045) and 1 h after incision (76.3 ± 13.0/min vs. 67.5 ± 9.3/min, *p* = 0.001), and after tourniquet deflation (80.6 ± 11.8/min vs. 66.9 ± 11.3/min, *p* < 0.001) (Figure 3A, Supplementary Table S1). The mean BP was significantly higher before (105.1 ± 12.6 mmHg vs. 97.0 ± 19.7 mmHg, *p* = 0.034) and after tracheal intubation (106.8 ± 19.4 mmHg vs. 87.0 ± 22.6 mmHg, *p* < 0.001) and 30 min (97.3 ± 13.0 mmHg vs. 85.0 ± 10.2 mmHg, *p* < 0.001) and 1 h after incision (99.6 ± 11.1 mmHg vs. 86.9 ± 10.8 mmHg, *p* < 0.001) (Figure 3B, Supplementary Table S2).

Emergence time in the OR was definitely faster in the RMMZ group than in the SEVO group, and the recovery time required to reach an Aldrete score of nine or higher was comparable between the two groups. Postoperative Cr levels were comparable between the two groups. The increase in postoperative Cr levels compared to pre-operative levels was also comparable between the RMMZ and SEVO groups. Furthermore, the postoperative HLOS was comparable between both the groups (Table 3).

In terms of POUR (26 (66.7%) in the RMMZ group vs. 28 (71.8%) in the SEVO group, *p* = 0.806), PONV (15 (38.5%) in the RMMZ group vs. 20 (51.3%) in the SEVO group, *p* = 0.362), PPC (6 (15.4%) in the RMMZ group vs. 5 (12.8%) in the SEVO group, *p* = 1.000), or delirium (5 (12.8%) in the RMMZ group vs. 2 (5.1%) in the SEVO group, *p* = 0.428) as postoperative complications within POD 2, these were comparable between the RMMZ and SEVO groups (Figure 2, Supplementary Table S3).

There was no definite correlation between LFT and extubation time in the RMMZ group as subgroup analysis (International Normalized Ratio and extubation time, *r* = 0.14, *p* = 0.403; Total Bilirubin, *r* = 0.07, *p* = 0.668; Aspartate Aminotransferase, *r* = 0.17, *p* = 0.300; and Alanine Aminotransferase, *r* = −0.22, *p* = 0.172) revealed (Supplementary Figure S1).

## 4. Discussion

This prospective, assessor-blinded, randomized controlled study evaluated the incidence of AKI after using RMMZ or SEVO as anesthesia maintenance agents in elderly patients undergoing TKA under general anesthesia. In terms of short-term postoperative complications, the AKI, POUR, PONV, PPC, and delirium rate were comparable between the RMMZ and SEVO groups. However, intraoperative HR and BP were higher and less variable in the RMMZ group than in the SEVO group. During surgery, inotropic administration was lower in the RMMZ group than in the SEVO group, and additional sedatives (midazolam) and opioids (remifentanil) were administered more often in the RMMZ group than in the SEVO group. The emergence time in the OR was shorter in the RMMZ group than that in the SEVO group.

Previous studies have shown that hemodynamics are more stable when RMMZ is administered than when conventional anesthetics, such as propofol or inhalational agents, are administered [15,16,17]. The current study was conducted with the hypothesis that hemodynamic stability affects the incidence of postoperative AKI; however, no difference was confirmed between the RMMZ and SEVO groups. In the RMMZ group, intraoperative hemodynamic stability and low inotropic requirements were confirmed again. The reason for the hemodynamic stability in the RMMZ group cannot be excluded from the high dose of opioids administered intraoperatively. However, the additional opioid infusion dose was within the recommended range on the remifentanil product label, and the overall BP was higher in the RMMZ group than in the SEVO group. Consequently, additional opioids were not administered excessively. In addition, when hypotension occurred in the SEVO group, BP did not decrease enough to cause AKI because of the active administration of inotropic agents.

The emergence time in the OR was faster in the RMMZ group than that in the SEVO group. Choi et al. [24] compared RMMZ and propofol and found that the time to tracheal extubation was approximately 600 s without reversal of flumazenil in the RMMZ group. Furthermore, the recovery times were comparable between the RMMZ and propofol groups. Song et al. [25] compared RMMZ and an inhalant agent and found that the time to tracheal extubation was approximately 860 s and 480 s in the RMMZ and inhalant agent groups, respectively. Furthermore, flumazenil was administered to approximately 6% of the participants in the RMMZ group. In the current study, flumazenil was infused into all participants in the RMMZ group, resulting in a faster recovery time than in the SEVO group. However, the time taken to fulfill the criteria for exit from the PACU was comparable between the RMMZ and SEVO groups. Compared to previous studies, the recovery time in the current study was reduced by approximately half with the administration of flumazenil.

The incidence of postoperative complications was comparable between the RMMZ and SEVO groups. However, the incidence of POUR was high in both groups. In a meta-analysis by Chang et al. [26], the incidence of POUR was approximately 10%, and age ≥50 years was a risk factor. In a previous trial by Cha et al. [27], the incidence of POUR was approximately 40%; however, POUR was defined as urine retention urine ≥400 mL. In the present study, the total incidence of POUR was relatively high compared with previous studies because the participants were elderly patients, and POUR was defined as retention urine ≥ 300 mL. Further research is required to reduce POUR in elderly patients undergoing TKA. The incidence of PONV was comparable between the RMMZ and SEVO groups; however, PONV tended to occur more frequently in the SEVO group than in the RMMZ group. Elbakry et al. [28] reported that PONV increased when inhalation was used compared to TIVA. A similar trend was observed in the current study. RMMZ could be the method to reduce PONV in a similar way to other TIVA drugs. The incidence of delirium tended to be higher in the RMMZ group than in the SEVO group. We considered that the incidence of delirium was slightly higher because RMMZ is a benzodiazepine; however, in previous studies, the correlation between RMMZ and delirium remained controversial [29,30]. Previous studies have demonstrated that RMMZ prevents emergent delirium in children [31]. Additional large-scale studies are required to evaluate these differences.

Stöhr et al. [32] reported that decreased liver function delayed recovery when RMMZ was used. In the subgroup analysis of the current study, the correlation between the LFT and emergence time was evaluated in the RMMZ group. There was no significant correlation. Most of the participants in this study had normal LFT results. Consequently, the recovery time did not change with small differences in liver function in the normal LFT group.

Another study drug, sevoflurane, reacted with some carbon dioxide absorbents to decompose into Compound A [33]. In studies conducted on rats, Compound A was reported to cause nephrotoxicity under low fresh gas flow conditions [34,35]. However, several human studies reported no association between compound A and renal toxicity [36,37]. In particular, in a relatively recently published large-scale retrospective study conducted by Park et al. [38] on non-cardiac surgery patients, the incidence of AKI was comparable between the SEVO group (2.3%, 257/11070) and the no-SEVO group (2.5%, 66/2631) (*p* = 0.57), and SEVO anesthesia was not related with postoperative AKI (OR 1.32; 95% CI: 0.94–1.86; *p* = 0.11). Similar to previous studies, the incidence of AKI in the present study was comparable between the RMMZ (12.8%) and SEVO (10.3%) groups (*p* = 1.000). The higher incidence of AKI compared to previous studies is considered to be because the present study was conducted with elderly patients older than 65 years.

### Limitations

This study had some limitations. First, because of the considerable differences between the administration methods of RMMZ and SEVO, double-blinding could not be achieved. However, an outcome assessor which was blinded (by the Y.N.A.) was used to minimize bias in the postoperative outcome evaluation.

Second, the intraoperative RMMZ was titrated against BIS. Given that the BIS is not an anesthetic depth monitoring system designed based on the RMMZ, it may not properly reflect the depth of anesthesia induced by the RMMZ. Consequently, additional sedatives were administered more frequently. However, most previous studies related to RMMZ adjusted the dose based on the BIS during surgery [39,40]. If RMMZ-based anesthesia depth monitoring equipment is developed in the future, the dose will be titrated more accurately.

Third, the participants were limited to elderly patients undergoing TKA, and most were relatively healthy (ASA I or II, 93.6%, 73/78) and female (85.9%, 67/78). However, the strength of this trial was the administration of RMMZ, which showed hemodynamic stability in elderly patients who were expected to have decreased BP during surgery. Additionally, when only TKA was included, the surgical position, operative time, operative difficulty, and blood loss were relatively consistent. In previous studies, most of the patients underwent TKA were female patients [41,42]. A study by Koh et al. [43] on Korean patients reported that the proportion of female participants was approximately 90%, which is comparable to the female sex ratio in the current study. Healthy female patients were evenly distributed in both groups; consequently, we minimized bias when analyzing the outcomes. Further studies should be conducted with even sex distribution in critically ill patients.

## 5. Conclusions

RMMZ may be recommended for patients who are anticipated to experience a decrease in intraoperative BP. However, stable hemodynamics with RMMZ are not sufficient to prevent AKI. Further large-scale, double-blind, randomized controlled trials of RMMZ should be performed.

## Figures and Tables

**Table 1:** Demographic data.

	RMMZ (*n* = 39)	SEVO (*n* = 39)	*p*-Value
Age (yr)	75 (71–78)	72 (69–78)	0.298
Female (*n*)	33 (84.6%)	34 (87.2%)	1.000
Body mass index (kg/m^2^)	24.3 (22.5–26.6)	23.6 (22.4–27.3)	0.826
ASA-PS class (I/II/III)	3 (7.7%) /33 (84.6%) / 3 (7.7%)	7 (17.9%)/30 (76.9%)/2 (5.1%)	0.379
Diabetes	13 (33.3%)	9 (23.1%)	0.450
Hypertension	32 (82.1%)	30 (76.9%)	0.779
Chronic kidney disease	4 (10.3%)	2 (5.1%)	0.671
Hematocrit (%)	39.7 (37.2–41.5)	39.5 (37.5–41.7)	0.877
Platelet (×10^3^/μL)	238 (199–263)	222 (205–249)	0.675
INR	0.99 (0.95–1.01)	0.96 (0.93–1.00)	0.111
Total bilirubin (mg/dL)	0.66 (0.54–0.87)	0.68 (0.57–0.80)	0.920
AST (IU/L)	24 (22–27)	25 (23–28)	0.228
ALT (IU/L)	19 (14–24)	19 (17–22)	0.779
Creatinine (mg/dL)	0.68 (0.57–0.85)	0.65 (0.56–0.75)	0.487

Data are presented as medians (interquartile range) or numbers (%). RMMZ: remimazolam; SEVO: sevoflurane; ASA-PS: American Society of Anesthesiologists Physical Status; INR: International Normalized Ratio; AST: Aspartate Aminotransferase; ALT: alanine aminotransferase.

**Table 2:** Intraoperative outcomes.

	RMMZ (*n* = 39)	SEVO (*n* = 39)	*p*-Value
RMMZ administered rate (μg/kg/h)	1.17 (0.99–1.52)	0	<0.001 *
Mean SEVO concentration (vol%)	0	2.1 (1.9–2.2)	<0.001 *
Remifentanil administered rate (μg/kg/min)	0.11 (0.08–0.14)	0.08 (0.05–0.11)	0.001 *
Inotropic administration (*n*)	6 (15.4%)	26 (66.7%)	<0.001 *
Vasodilator administration (*n*)	33 (84.6%)	8 (20.5%)	<0.001 *
Sedative administration (*n*)	8 (20.5%)	0 (0.0%)	0.009 *
Anesthesia time (min)	145 (140–155)	150 (140–160)	0.740
Surgical time (min)	105 (97.5–115)	105 (95–110)	0.948
Fluid infusion (mL)	400 (300–450)	350 (300–400)	0.362
Estimated blood loss (mL)	30 (30–50)	30 (25–50)	0.913

Data are presented as numbers (%) or medians (interquartile range). * Statistical significance. RMMZ: remimazolam; SEVO: sevoflurane.

**Table 3:** Postoperative outcomes.

	RMMZ (*n* = 39)	SEVO (*n* = 39)	*p*-Value
Emergence time			
Eye open (s)	225 (162–380)	372 (312–496)	0.001 *
Extubation (s)	348 (215–442)	428 (336–570)	0.006 *
Exit the OR (s)	392 (252–561)	479 (386–648)	0.008 *
Aldrete score ≥ 9 (min)	29.7 (27.0–36.1)	31.0 (28.6–34.2)	0.269
Postoperative Cr (mg/dL)	0.85 (0.74–0.96)	0.85 (0.78–1.00)	0.869
Postop Cr—Preop Cr	0.19 (0.10–0.25)	0.22 (0.15–0.26)	0.151
Postoperative HLOS (d)	8 (6–13)	7 (6–13)	0.932

Data are presented as median (interquartile range). * Statistical significance. RMMZ: remimazolam; SEVO: sevoflurane; Cr: Creatinine; HLOS: hospital length of stay.

## Data Availability

The datasets analyzed during the study are available from the corresponding author upon reasonable request. The datasets are not publicly available because of privacy or ethical restrictions.

## Supplementary Materials

The following supporting information can be downloaded at: https://www.mdpi.com/article/10.3390/jpm13050789/s1, Table S1: Heart rate at different perioperative phages; Table S2: Blood pressure at different perioperative phages; Table S3: Incidence of postoperative complications; Figure S1: Correlation between tracheal extubation time and INR (A), Total Bilirubin (B), AST (C), and ALT (D).
