# Supplementary material for: Comparison of the Incidence of Postoperative Acute Kidney Injury Following the Administration of Remimazolam or Sevoflurane in Elderly Patients Undergoing Total Knee Arthroplasty: A Randomized Controlled Trial
Source: J Pers Med. 2023 May 1;13(5):789. doi: 10.3390/jpm13050789 (PMC10223479; doi:10.3390/jpm13050789)
Supplement: Supplementary file 1 [file jpm-13-00789-s001.zip › supplementary table 1 2 3_RMMZ AKI.pdf]

Table S1. Heart rate at different perioperative phages.

	RMMZ (n=39)	SEVO (n=39)	*p*-Value
Initial	76.9 ± 12.8	73.2 ± 11.2	0.176
Before intubation	81.6 ± 11.3	78.9 ± 13.8	0.340
3 min after intubation	86.0 ± 13.1	78.8 ± 13.9	0.021*
Skin incision	82.1 ± 13.9	81.7 ± 12.5	0.898
30 min after incision	77.2 ± 12.7	71.8 ± 10.7	0.045*
1 h after incision	76.3 ± 13.0	67.5 ± 9.3	0.001*
Tourniquet deflation	80.6 ± 11.8	66.9 ± 11.3	< 0.001*
PACU	79.3 ± 15.5	76.1 ± 12.8	0.319

Data denote means ± standard deviations. * Statistical significance. RMMZ, remimazolam; SEVO, sevoflurane; PACU, post-anesthetic care unit.

Table S2. Blood pressure at different perioperative phages.

	RMMZ (n=39)	SEVO (n=39)	*p*-Value
Systolic blood pressure			
Initial	160.5 ± 18.7	160.8 ± 22.5	0.957
Before intubation	145.2 ± 17.8	131.3 ± 27.9	0.011*
3 min after intubation	145.2 ± 28.2	117.8 ± 21.6	< 0.001*
Skin incision	158.8 ± 19.1	155.6 ± 20.5	0.473
30 min after incision	134.9 ± 17.0	116.6 ± 14.6	< 0.001*
1 h after incision	140.2 ± 14.5	118.3 ± 14.8	< 0.001*
Tourniquet deflation	114.9 ± 19.0	106.7 ± 17.3	0.050
PACU	157.3 ± 19.8	157.4 ± 28.2	0.993
Mean blood pressure			
Initial	111.2 ± 12.7	106.6 ± 15.8	0.165
Before intubation	105.1 ± 12.6	97.0 ± 19.7	0.034*
3 min after intubation	106.8 ± 19.4	87.0 ± 22.6	< 0.001*
Skin incision	115.8 ± 15.4	114.1 ± 14.9	0.618
30 min after incision	97.3 ± 13.0	85.0 ± 10.2	< 0.001*
1 h after incision	99.6 ± 11.1	86.9 ± 10.8	< 0.001*
Tourniquet deflation	81.5 ± 14.0	78.1 ± 12.8	0.273
PACU	110.8 ± 12.9	109.4 ± 17.3	0.685
Diastolic blood pressure			
Initial	88.4 ± 11.4	86.1 ± 11.5	0.360
Before intubation	85.8 ± 12.4	80.0 ± 14.8	0.064
3 min after intubation	87.4 ± 17.9	75.6 ± 18.9	0.006*
Skin incision	94.5 ± 12.5	97.3 ± 13.4	0.345
30 min after incision	78.5 ± 11.7	71.4 ± 8.8	0.003*
1 h after incision	80.1 ± 10.9	72.4 ± 8.7	0.001*
Tourniquet deflation	65.0 ± 11.7	63.6 ± 11.0	0.578
PACU	86.5 ± 11.8	85.0 ± 13.8	0.610

Data denote means ± standard deviations. * Statistical significance. RMMZ, remimazolam; SEVO, sevoflurane; PACU, post-anesthetic care unit.

Table S3. Incidence of postoperative complications.

	RMMZ (n=39)	SEVO (n=39)	*p*-Value
Postoperative kidney injury	5 (12.8%)	4 (10.3%)	1.000
Postoperative urinary retention	26 (66.7%)	28 (71.8%)	0.806
Postoperative nausea vomiting	15 (38.5%)	20 (51.3%)	0.362
Postoperative pulmonary complication	6 (15.4%)	5 (12.8%)	1.000
Delirium	5 (12.8%)	2 (5.1%)	0.428

Data denote numbers (%). RMMZ, remimazolam; SEVO, sevoflurane.
