# Supplementary figures and images for: Comparison of the Incidence of Postoperative Acute Kidney Injury Following the Administration of Remimazolam or Sevoflurane in Elderly Patients Undergoing Total Knee Arthroplasty: A Randomized Controlled Trial
Source: J Pers Med. 2023 May 1;13(5):789. doi: 10.3390/jpm13050789 (PMC10223479; doi:10.3390/jpm13050789)

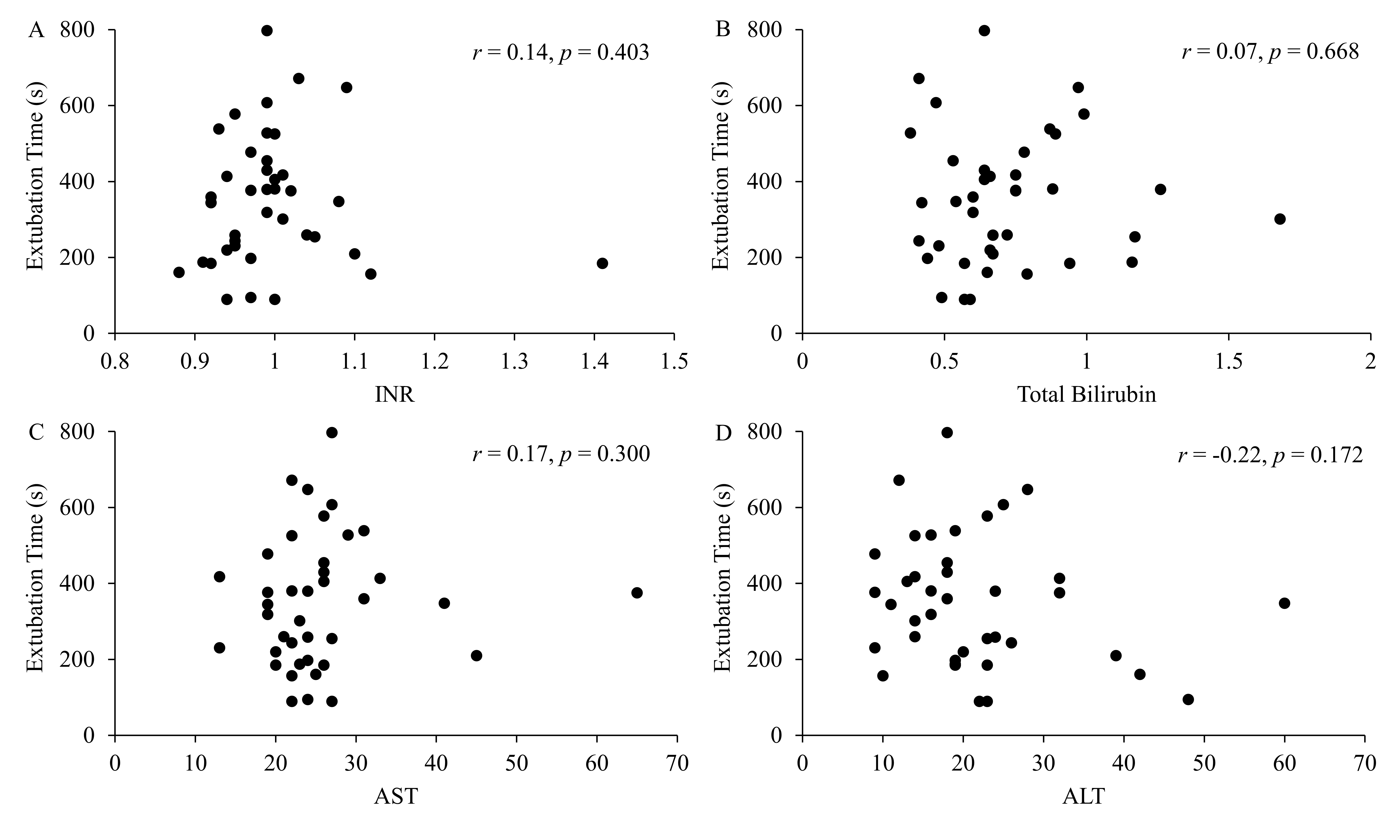

Supplement: Supplementary file 1 [file jpm-13-00789-s001.zip › supplementary figure S1 LFT extu RMMZ AKI.tif]
